# Immunostimulatory Effects Triggered by *Enterococcus faecalis* CECT7121 Probiotic Strain Involve Activation of Dendritic Cells and Interferon-Gamma Production

**DOI:** 10.1371/journal.pone.0127262

**Published:** 2015-05-15

**Authors:** Matías Alejandro Molina, Ailén Magalí Díaz, Christina Hesse, Wiebke Ginter, María Virginia Gentilini, Guillermo Gabriel Nuñez, Andrea Mercedes Canellada, Tim Sparwasser, Luciana Berod, Marisa Silvia Castro, Marcela Alejandra Manghi

**Affiliations:** 1 Laboratorio de Modulación de la Respuesta Inmune, IDEHU (CONICET-UBA). Ciudad Autónoma de Buenos Aires, Argentina; 2 Institute of Infection Immunology, TWINCORE, Centre for Experimental and Clinical Infection Research, a Joint Venture between the Medical School Hanover (MHH) and the Helmholtz Centre for Infection Research (HZI), Hannover, Germany; 3 Instituto de Inmunología, Genética y Metabolismo, INIGEM (CONICET-UBA). Ciudad Autónoma de Buenos Aires, Argentina; 4 Laboratorio de Anticuerpos Asimétricos e Inmunología de la Reproducción, IDEHU (CONICET-UBA). Ciudad Autónoma de Buenos Aires, Argentina; Institut National de la Santé et de la Recherche Médicale (INSERM), FRANCE

## Abstract

Probiotics can modulate the immune system, conferring beneficial effects on the host. Understanding how these microorganisms contribute to improve the health status is still a challenge. Previously, we have demonstrated that *Enterococcus faecalis* CECT7121 implants itself and persists in the murine gastrointestinal tract, and enhances and skews the profile of cytokines towards the Th1 phenotype in several biological models. Given the importance of dendritic cells (DCs) in the orchestration of immunity, the aim of this work was to elucidate the influence of *E*. *faecalis* CECT7121 on DCs and the outcome of the immune responses. In this work we show that *E*. *faecalis* CECT7121 induces a strong dose-dependent activation of DCs and secretion of high levels of IL-12, IL-6, TNFα, and IL-10. This stimulation is dependent on TLR signaling, and skews the activation of T cells towards the production of IFNγ. The influence of this activation in the establishment of Th responses *in vivo* shows the accumulation of specific IFNγ-producing cells. Our findings indicate that the activation exerted by *E*. *faecalis* CECT7121 on DCs and its consequence on the cellular adaptive immune response may have broad therapeutic implications in immunomodulation.

## Introduction

The gastrointestinal microbiota varies in a continuum range between mutualism and pathogenicity, as a consequence of both residential and ingested microorganisms [1]. This microbiota is essential to human health and imprints unique characteristics in each human being; it contributes to food digestion and the development and optimal functioning of the immune system. Moreover, a disproportion of these microorganisms has not only local but also systemic consequences, as observed in cases of intestinal inflammation and infection [1]. Therefore, maintenance of the correct equilibrium and even an enhancement of the beneficial effects of the microbiota offer an opportunity for treatments both in health and disease conditions.

In the last years, the increased interest in the beneficial functions of the human microflora has resulted in the selection of specific species with putative health-promoting capacities. These selected microorganisms, recognized as probiotics, are defined as “live microorganisms which when administered in adequate amounts confer a health benefit on the host” [2]. Clinical applications of probiotics include the prevention and treatment of gastrointestinal infections, inflammatory bowel diseases, allergies, and also their use as vaccine adjuvants [1]. The mechanisms by which probiotics exert these beneficial effects are diverse and can be classified into three main categories: (1) those involving the influence on other microorganisms (especially pathogens); (2) those by which the barrier function played by the intestinal epithelium is enhanced; and (3) those involving the modulation of the immune system. While the first one is the best-studied effect, the immunomodulatory mechanisms are less understood and seem to be genus/strain specific [1]. Understanding how these microorganisms contribute to improve the health status is still a challenge, and a better knowledge of how probiotic bacteria interact with host cells is needed for their optimized application.

Dendritic cells (DCs) include a heterogeneous population of cells whose central function is to present antigens. In contrast to other antigen-presenting cells, DCs have the unique capacity of stimulating primary immune responses. Besides, they can induce either immunogenic or tolerogenic immune responses depending upon the type of DC and the nature of the antigen encountered [3–7]. DCs recognize and respond to microbial structures via pattern-recognition receptors (PRRs) including Toll-like receptors (TLRs) and lectins, among others [8–9]. Recognition of pathogen-associated molecular patterns (PAMPs) by these receptors results in functional changes on DCs including up-regulation of their migratory capacity, expression of co-stimulatory molecules, and cytokine production [10–11]. Different stimuli induce the production of specific cytokines scenarios that are responsible for the fine-tuning of an adequate immune response in each case. Interleukin-(IL-)12-producing DCs are responsible for the shift of the adaptive immune response towards a T helper (Th) 1-profile. IL-6 and TNFα are pro-inflammatory cytokines that have important effects in systemic inflammation. In contrast, IL-10 results in anti-inflammatory actions and is important for the generation of regulatory T cells. Despite all the available information about the understanding of the general functions of DCs, the mechanisms by which DCs respond to probiotics in the intestine and select appropriate immune responses have been poorly studied [1].

Most microorganisms that are considered probiotics are generally selected from the *Lactobacillus* or *Bifidobacterium* genera [1]. Other probiotics that belong to the Gram-positive Lactic Acid Bacteria include *Enterococcus faecalis* strains, and even Gram-negative microorganisms such as *Escherichia coli* Nissle 1917 or yeasts identified as *Saccharomyces boulardii* have also been demonstrated to confer beneficial effects to the host [1].

*Enterococcus faecalis* CECT7121 is a promising candidate for being considered as a probiotic strain [12–15]. This microorganism has been recovered from a corn silage at an establishment located in the city of Tandil (Argentina). Employing several murine models, we have previously demonstrated that *E*. *faecalis* CECT7121 implants itself and persists in the gastrointestinal tract [12], and enhances and skews the profile of cytokines to the Th1 phenotype in situations such as vaccination, anti-tumoral immunity, and allergic reactions [13–15]. In these models, we have shown that the intragastric administration of *E*. *faecalis* CECT7121 can induce a stronger cellular anti-tetanic and anti-diphtheric response in BALB/c mice immunized with Diphtheria-Tetanus-*Bordetella pertussis* vaccine [13]. This probiotic is also able to reduce the mortality of animals challenged with a lethal dose of the murine LBC lymphoma cells, triggering mechanisms that include the generation of a Th1 response characterized by a high production of IL-12 and interferon-gamma (IFNγ) [14]. Finally, this bacterium prevents the establishment of an allergic response against ovalbumin, diminishing Immunoglobulin-E (IgE) specific titers as well as Th2 cytokines levels (i.e. IL-4, IL-5, and IL-13), and impairing the generation of active cutaneous anaphylactic reactions [15].

In the present work, we intended to elucidate the main mechanisms that are triggered by *E*. *faecalis* CECT7121 and that lead to the beneficial effects observed in mice. In this context, we analyzed the effects caused by the stimulation of DCs and the influence of this activation in the establishment of Th responses. In addition, we studied the role of regulatory cells in the beneficial effects conferred by *E*. *faecalis* CECT7121. Our results allowed us to conclude that the activation of DCs might be the most relevant consequence after inoculation of this probiotic, skewing the adaptive immune response towards the production of IFNγ. This work postulates *E*. *faecalis* CECT7121 as a novel adjuvant candidate for mucosal immunostimulation, in both oral and systemic vaccination strategies.

## Materials and Methods

### Ethical statement

All animal experiments were performed in compliance with the Argentinean and German (TierSchG BGBl. I S. 1105; 25-05-1998) animal protection laws. Mice were housed and handled in accordance with good animal practices as defined by the National Research Council of the National Academies (USA), the Federation of European Laboratory Animal Science Associations (FELASA, European Union), and the national animal welfare body GV-SOLAS (Germany). All animal experiments were approved by the Instituto de Estudios de la Inmunidad Humoral “*Prof*. *Dr*. *Ricardo A*. *Margni*” (IDEHU, CONICET-UBA) and the Lower Saxony Committee on the Ethics of Animal Experiments, as well as the responsible state office (Lower Saxony State Office of Consumer Protection and Food Safety) under the permit numbers 33.9-42502-04-11/0453 considering the German Animal Welfare Act and UBACyT B028 (2008–2011) considering Argentinean institutions. All procedures were performed after mice were euthanized by CO_2_ or cervical dislocation, and every effort was made to minimize animal suffering.

### Animals

Six- to eight-week-old BALB/c and C57BL/6 wild-type mice were obtained from the Animal Facility at the *Facultad de Ciencias Veterinarias* (Universidad de Buenos Aires, Argentina) or at the *Helmholtz-Zentrum für Infektionsforschung* (HZI, Germany), and were kept at the Animal Facility of the *Instituto de Estudios de la Inmunidad Humoral* (CONICET-UBA, Argentina) or at the *Twincore-Zentrum für Infektionsforschung* (MHH-HZI, Germany), respectively.

Human DC-SIGN (dendritic cell-specific intercellular adhesion molecule-3-grabbing non-integrin, CD209) transgenic mice, termed “hSIGN”, were generated using the murine CD11c promoter driving expression of a human DC-SIGN cDNA sequence as previously described [16]. Myeloid differentiation primary response gene 88 knock-out (MyD88^-/-^) [17], mannose receptor knock-out (MR^-/-^) [18], and OT-II mice [19] were also employed. hSIGN × MyD88^-/-^ and hSIGN × MR^-/-^ mice were crossed at HZI (Germany). All genetically modified mice belonged to the C57BL/6 genetic background, and were kept at *Twincore* (MHH-HZI, Germany).

All animals were housed (n = 5 mice/cage) under specific conditions according to the “Guide for the Care and Use of Laboratory Animals” (National Research Council of the National Academies, USA), with controlled air temperature (20–22°C), humidity, and 12 h light/dark cycles; food and water were provided *ad libitum*.

### *Enterococcus faecalis* CECT7121 suspensions and sub-cellular fractions

*E*. *faecalis* CECT7121 was grown in Triptone Soy Broth (Biokar Diagnostics, France) at 37°C for 18 h. After incubation, the culture was harvested by centrifugation at 5,800 *g* for 15 min (4°C) and washed with sterile phosphate buffered saline (PBS). *E*. *faecalis* CECT7121 suspensions were prepared as previously described (approximately 3.0×10^8^ CFU/mL in sterile PBS) [12]. For *in vitro* studies, heat-killed microorganisms (Ef∅; 80°C, 1 h) were used to stimulate cell cultures [12–15]. Identification and contamination/inactivation were routinely controlled.

Cell walls and soluble lysate fractions from *E*. *faecalis* CECT7121 were obtained by bacterial disruption according to a modified protocol from Araki *et al* [20]. Briefly, *E*. *faecalis* CECT7121 was grown in 1–2 L of TSB, during 18 h at 37°C and continuous shaking (150 rpm). Bacterial suspensions were then washed with sterile PBS between steps (6,000 *g*, 20 min, 4°C), and subjected to different treatments: (1) sonication (3 cycles × 5 min, power output 12 W (Ultrasonic Processor GE 50; Sonics & Materials, Inc., USA), in cold, sterile, distilled water); (2) osmotic lysis (cold 1 M NaCl (Biopack, Argentina), 10 min); (3) boiling (20 min, sterile, distilled water); (4) trypsin digestion (2 mg (Gibco, USA) in 50 mM phosphate buffer, pH 7.50, 2 h, 37°C); (5) SDS treatment (0.4% (w/v) SDS (Gibco, USA), 1 h at room temperature). Between each step, bacterial suspension was centrifuged (20,000 *g*, 30 min-1 h, 4°C). The “soluble lysates” were collected after every cycle of sonication, clarified by centrifugation (20,000 *g*, 1 h), filtered (0.22 μm), lyophilized, and kept at -20°C. “Bacterial cell walls” were collected by differential centrifugation (2,700 *g*, 15 min; and 20,000 *g*, 30 min) after the last centrifugation step; the pellet was resuspended in sterile distilled water, lyophilized, and kept at -20°C.

Lipoteichoic acid (LTA) was purified following the improved procedure described by Morath and colleagues [21]. A defrosted aliquot of bacteria was treated with an equal volume of *n*-butanol (Biopack, Argentina) under stirring for 30 min at room temperature. After centrifugation (13,000 *g* for 20 min), the aqueous phase was lyophilized, resuspended with chromatography start buffer (15% *n*-propanol in 0.1 M ammonium acetate, pH 4.70; both from Biopack, Argentina), and centrifuged at 45,000 *g* for 15 min. The supernatant was subjected to hydrophobic interaction chromatography on Octyl Sepharose 4 Fast Flow (GE Healthcare Life Sciences, USA) and the elution was performed by increasing the percentage of *n*-propanol in the acetate buffer up to 60% (linear gradient). Purified LTA was detected by Western Blot employing specific rabbit antiserum against *E*. *faecalis* CECT7121 cell wall fraction (obtained in our laboratory).

### Generation and *in vitro* stimulation of mouse Bone Marrow-derived Dendritic Cells (BM-DCs)

*In vitro* generation of Granulocyte-Monocyte Colony Stimulatory Factor (GM-CSF)- driven DCs from Bone Marrow (BM) precursors has been described previously [22]. Briefly, BM cells were isolated from femurs and tibiae, and red blood cells (RBC) were lysed at room temperature by standard procedures employing hypotonic RBC lysis buffer (150 mM NH_4_Cl, 10 mM NaHCO_3_, 0.1 mM EDTA, pH 7.40; all from Biopack, Argentina) [23]. Then, 2.0–4.0×10^6^ BM cells were seeded onto bacterial sterile Petri dishes with 10–20 mL of complete RPMI 1640 culture medium (cRPMI) containing 10% (v/v) fetal calf serum (FCS), 100 IU/mL penicillin, 100 μg/mL streptomycin, 300 μg/mL L-glutamine, 10 mM HEPES, and 50 μM 2-mercaptoethanol (all from Gibco, USA, with the exception of FCS: Natocor, Argentina or Hyclone, USA), plus 10% GM-CSF conditioned medium (prepared in our laboratory with GM-CSF present in culture supernatants of J588L cells). Cells were cultured for 6 to 8 d in cRPMI at 37°C and 5% CO_2_ before stimulation experiments were performed.

Immature BM-DCs (4.0×10^5^ DCs/mL) were stimulated for 0, 6, 12, 24, and 48 h (37°C, 5% CO_2_) with Ef∅ (MOI 1:1, 10:1, and 100:1) or with *Escherichia coli* O111:B4 lipopolysaccharide (LPS 1 μg/mL; Sigma-Aldrich, USA). A period of stimulation of 24 h was selected as the most suitable. The same amount of BM-DCs was stimulated for 24 h (37°C, 5% CO_2_) with bacterial cell walls (1, 10, 100, and 1,000 μg/mL), soluble lysates (1, 10, 100, and 1,000 μg of total proteins/mL), or purified LTA (1, 10, 100, and 1,000 μg/mL).

After stimulation, culture supernatants were collected and stored at -80°C, until different cytokines were evaluated by ELISA (IL-6, TNFα, IL-12p40, and IL-10). Stimulated cells were washed and resuspended employing cold sterile PBS plus 5 mM EDTA (5–10 min on ice), and stained for flow cytometry analysis: CD11c-APC (HL3), Major Histocompatibility Complex (MHC) class II (I-E[k])-PE (14-4-4S), CD80-FITC (16-10A1), CD40-PE (3/23), and CD83-PE (Michel-19); all from BD Biosciences (USA).

BM precursors from genetically modified animals (hSIGN, MyD88^-/-^, MR^-/-^, hSING × MyD88^-/-^, and hSIGN × MR^-/-^) were also employed for the generation of BM-DCs. Differentiation, stimulation, and analysis were performed as described above.

### DC-T cell co-culture under stimulation with *E*. *faecalis* CECT7121

GM-CSF derived BM-DCs were generated as previously described. On day 7, 2.5×10^4^ BM-DCs were incubated with 30 μg/mL ovalbumin (OVA, grade VI; Sigma-Aldrich, USA) for 2–3 h, washed and further incubated with different MOIs of Ef∅ overnight. Then, stimulated BM-DCs were washed and co-cultured for 4 days at 37°C and 5% CO_2_ with 5.0–10.0×10^4^ OT-II CD4^+^ T lymphocytes. OT-II CD4^+^ T cells were enriched by negative magnetic sorting using the Dynabeads Untouched Mouse CD4 Cells kit (Invitrogen, USA) following the manufacturer’s protocol. On day 4, cell suspensions (5.0–10.0×10^6^ cells) were stimulated during 6 h with 0.1 μg/mL phorbol 12-myristate 13-acetate (PMA) and 1 μg/mL Ionomycin (both from Sigma-Aldrich, USA); 3 μg/mL Brefeldin A (eBioscience, USA) were added for the last 2 h of incubation, before staining and data acquisition.

### Spleen and mesenteric lymph nodes cell cultures

For *ex vivo* cultures, aseptically removed spleens and mesenteric lymph nodes (MLNs) were cut into pieces and cell suspensions were passed through a 200 μm mesh nylon sieve. Cells were centrifuged (10 min, 200 *g*, 4°C) and pellets were washed with sterile PBS. After the RBC lysis, cells were counted in a Neubauer’s haemocytometer by Trypan dye blue exclusion. One hundred microlitres of spleen or MLNs cell suspensions (4.0×10^6^ cells/mL) were cultured with the same volume of cRPMI alone or Ef∅ (MOI 10:1) in quadruplicates at 37°C and 5% CO_2_ in air. Concanavalin A (ConA, 10 μg/mL, 100 μL; Vector Labs, USA) was used as mitogen. In each experiment, two cell cultures were performed: one to assess cell proliferation and the other to determine the cytokine levels in supernatants. After 72 h, supernatants were collected and stored at -80°C until assayed for cytokine levels. Proliferative responses were assessed by [^3^H]-thymidine (PerkinElmer Inc., USA) uptake employing a Liquid Scintillation Analyzer 1600TR (Packard, USA). Proliferation results were expressed as mean counts per minute (cpm) ± SEM of quadruplicate cultures.

### Identification of immune cellular populations by flow cytometry

Cellular suspensions from spleen or MLNs were specifically stained with labeled antibodies for flow cytometry assays. Antibodies (and clones) employed were: CD3ε -APC (145-2C11), CD19-Pacific Blue (eBio1D3), CD11b-APC.Cy7 (M1/70), CD45/B220-PE (RA3-6B2), CD4-eFluor 450 (RM4-5), Forkhead box P3 (Foxp3)-PE (FJK-16s), CD11b-eFluor 450 (M1/70), and Gr-1-Alexa647 (RB6-8C5), all from eBioscience (USA); CD11c-Texas Red (N418) from Life Technologies (USA); and Ly6C-PE.Cy7 (HK1.4) from BioLegend (USA). For IFNγ intracellular staining, cells were stained employing CD4-APC (GK1.5), CD8α-eFluor 450 (53–6.7), and IFNγ-PE (XMG1.2), all from eBioscience (USA).

For the standard staining protocol, approximately 1.0×10^6^ cells were washed in PBS and stained with the LIVE/DEAD Fixable Aqua Dead Cell Stain Kit (Invitrogen, USA) to exclude dead cells. Cells were then washed with PBS and incubated in staining buffer (0.25% (w/v) bovine seroalbumin (BSA) (Sigma-Aldrich, USA) and 2 mM EDTA in PBS) containing Fc-block (αCD16/32, 2.4G2) for 10 min on ice. Cells were stained with labeled antibodies. In most cases, staining was performed for 20 to 30 min on ice and cells were fixed with 2% paraformaldehyde in PBS for 20 min on ice. Intracellular staining for Foxp3 was performed after fixing and permeabilizing the cells with the Foxp3 Fixation/Permeabilization Kit (eBioscience, USA). For intracellular staining of IFNγ, cell suspensions (5.0–10.0×10^6^ cells) were restimulated with PMA + Ionomycin as previously described. Suspensions were stained for exclusion of dead cells, then washed with PBA-S permeabilization buffer (PBA plus 0.5% saponin; Sigma-Aldrich, USA) and specifically stained for 20 min on ice.

Samples were stored at 4°C and acquired either on a BD FACSAria II (BD Biosciences, USA) or a CyAn ADP (Beckman Coulter, Inc., USA) flow cytometers. Data were analyzed using the FlowJo software version 9.3.2 (Tree Star, Inc., USA). Single stains and “fluorescence minus one” controls were used for accurate gating and compensation. Non-specific binding was estimated by isotype controls and cellular aggregates were excluded by SSC-W. Quantitative results were expressed as mean percentage ± SEM.

### Determination of cytokine levels by ELISA

BD OptEIA ELISA kits (BD Biosciences, USA) were employed to evaluate all cytokines with the exception of IL-13, which was detected by Mouse IL-13 Cytoset (Invitrogen, USA). Assays were carried out according to the manufacturer’s instructions. Results were expressed as mean concentration ± SEM of animals from the same experimental group.

### *In vivo* stimulation with probiotic-pulsed DCs

Approximately 4.0×10^6^ immature BM-DCs (C57BL/6 wild-type) were *in vitro* stimulated with cRPMI (DC) or Ef∅ (MOI 10:1; Ef-DC). After 18 h of incubation, stimulated BM-DCs were washed with sterile PBS and 5.0x10^5^ BM-DCs/mouse were intraperitoneally (i.p.) injected into naïve C57BL/6 mice [24].

Seven days after inoculation, animals were sacrificed, spleens were removed, and 1.0×10^6^ cells were cultured in the presence of cRPMI or Ef∅ (MOI 10:1) for 72 h. Cytokines (IFNγ, IL-13, and IL-10) were measured by ELISA in culture supernatants, as previously described [25–27].

### Intragastric treatment of mice with *Enterococcus faecalis* CECT7121

Sex-matched BALB/c mice (n = 5–10/group) were intragastrically (i.g.) inoculated with either sterile PBS (0.2 mL/mouse) or live *E*. *faecalis* CECT7121 (3.0×10^8^ CFU/mL, 0.2 mL/mouse) employing a non-flexible stainless steel animal feeding needles (20G x 1.5" with a 2.25 mm spherical tip, Thomas Scientific, USA). Inoculations were performed during 3 consecutive days, as previously described [12]. Animals were sacrificed and analyzed on days 4 and 11 post-last inoculation. The i.g. administration of *E*. *faecalis* CECT7121 did not cause any physical harm to the animals. In this work, none of the animals died as a result of treatment, nor showed signs of discomfort or illness such as weight loss, apathy, trembling, or restricted motility. The technical staff was responsible for monitoring the physical condition of the animals twice a day.

### Statistical analysis

*In vitro* and *in vivo* experiments were performed between 2 to 5 times. All values were presented as means with their standard errors. Statistical significance was evaluated using unpaired *t* tests or ANOVA, according to the experimental design. When variables had a normal distribution (D’Agostinos & Pearson’s omnibus normality test) and showed homoscedasticity (F test or Bartlett’s test to compare variances), parametric tests were employed: unpaired Student’s *t* test or one-way ANOVA plus Dunnett’s Multiple Comparison *post hoc* tests. Welch’s correction (unpaired *t* tests), or Log(Y) and sqrt(Y) transformations (ANOVA) were occasionally used when variables showed heteroscedasticity. When samples did not have a normal distribution or did not show homoscedasticity, non-parametric tests were assayed: Mann-Whitney U-test or Kruskal-Wallis ANOVA plus Dunn’s *post hoc* tests. Graphical and statistical analyses were performed with GraphPad Prism 5.0d for Mac OS X (GraphPad Software, USA). Values were considered significantly different at *p<0.05, **p<0.01, or ***p<0.001.

## Results

### *E*. *faecalis* CECT7121 activates BM-DCs *in vitro* and stimulates the production of both pro- and anti-inflammatory cytokines

**Fig 1:**
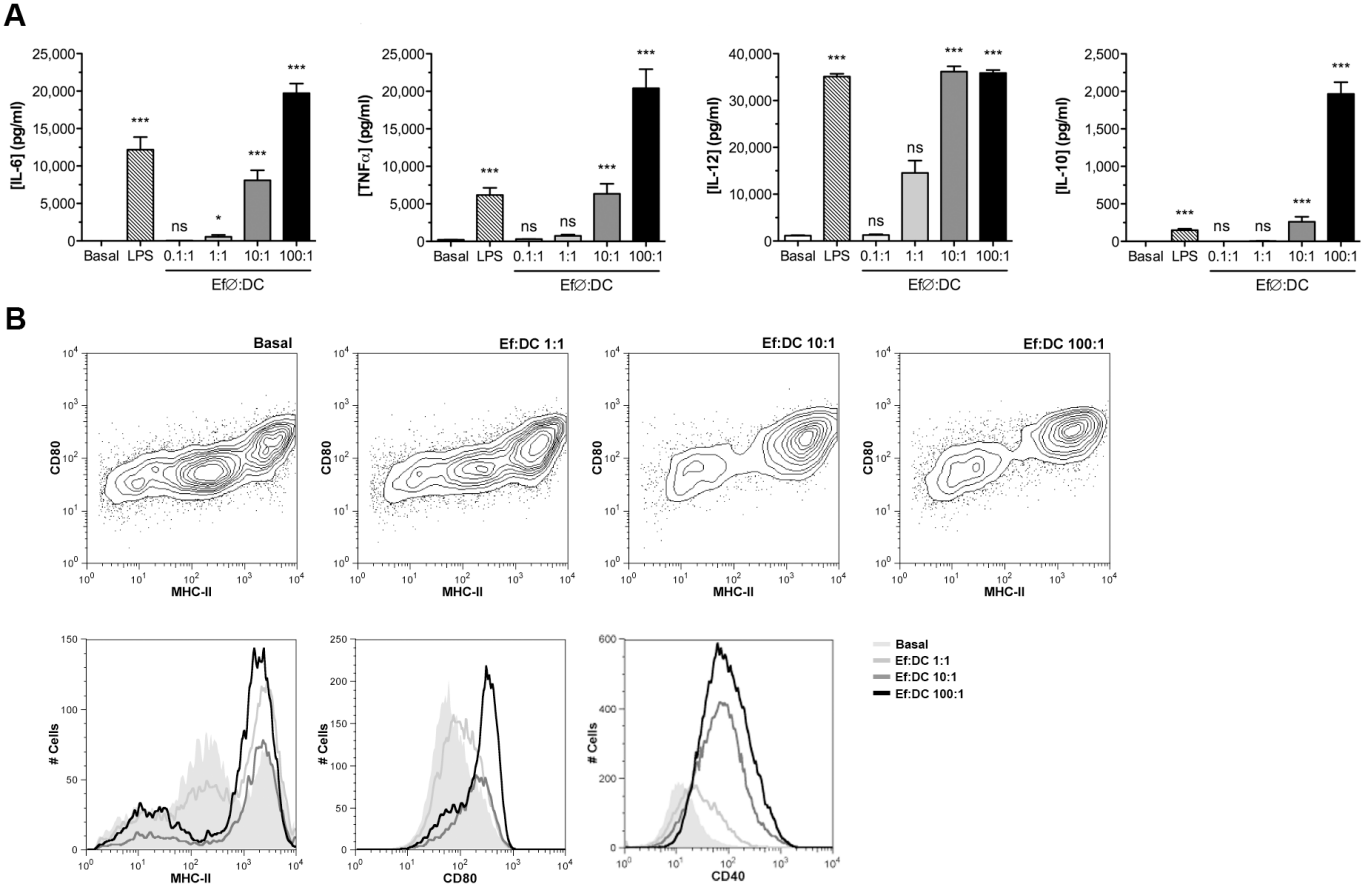
*In vitro* activation and cytokine production of BM-DCs by stimulation with *E*. *faecalis* CECT7121. (A) BALB/c BM-DCs were *in vitro* stimulated with heat-killed *E*. *faecalis* CECT7121 (Ef∅ at different MOIs), and IL-6, TNFα, IL-12, and IL-10 were measured in culture supernatants after 24 h. LPS (*E*. *coli* O26:B6) was employed as a positive control. Cytokine levels were expressed as mean pg/mL ± SEM. Comparisons were performed between Basal and stimulated cells. ns: non-significant, *p<0.05, ***p<0.001 (Kruskal-Wallis ANOVA and Dunn’s Multiple Comparison test). (B) The surface expression of MHC-II, CD80, and CD40 was assessed by flow cytometry after 24 h of BM-DCs stimulation with heat-killed *E*. *faecalis* CECT7121 (Ef∅). Results are representative from 5 different *in vitro* experiments. Heat-killed *E*. *faecalis* CECT7121 probiotic strain induce a strong dose-dependent secretion of cytokines and expression of activation markers on BM-DCs.

Given the importance of dendritic cells (DCs) in the initiation and organization of the immune response, BM-DCs were *in vitro* stimulated with Ef∅ at different time-points and the levels of IL-6, TNFα, IL-12, and IL-10 were determined by ELISA. *E*. *faecalis* CECT7121 induced the activation of BM-DCs and the production of all analyzed cytokines in a dose-dependent manner (Fig 1). When stimulated BM-DCs were analyzed by flow cytometry, an up-regulation of MHC-II, CD80, and CD40 was observed (Fig 1); additionally, an increase in CD83 expression was detected compared to non-stimulated cells (data not shown). These results clearly demonstrated that this probiotic bacterium is able to activate BM-DCs, not only by inducing the expression of MHC-II and co-stimulatory molecules and the production of pro-inflammatory cytokines, but also of IL-10 as a regulatory interleukin.

**Fig 2:**
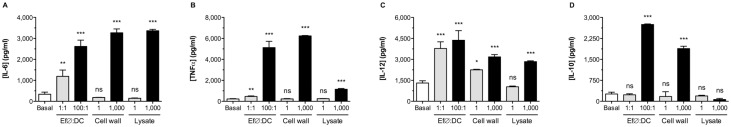
BM-DCs stimulation with *E*. *faecalis* CECT7121 cell walls and soluble lysate. BM-DCs were *in vitro* stimulated with *E*. *faecalis* CECT7121 cell walls (μg) and soluble lysate (μg of total protein), and IL-6, TNFα, IL-12, and IL-10 were measured in culture supernatants after 24 h (A, B, C, and D, respectively). Heat-killed *E*. *faecalis* CECT7121 (MOIs 1:1 and 100:1) was employed as a positive control. Cytokine levels were expressed as mean pg/mL ± SEM. ns: non-significant, *p<0.05, **p<0.01, ***p<0.001 (One-way ANOVA and Dunnett’s Multiple Comparison tests after ‘sqrt(IL-12)’ or ‘Log(TNFα)’ transformations). *E*. *faecalis* CECT7121 cell walls and soluble lysate show different behavior. Both stimuli are able to induce IL-12 and IL-6, but the soluble lysates induce low TNFα and fail to induce IL-10 production.

In order to identify the subcellular fractions involved in such activation, *E*. *faecalis* CECT7121 was dissected into bacterial cell walls and soluble lysates. Both stimuli activated BM-DCs *in vitro* in a dose-dependent manner, as determined by flow cytometry (data not shown) and cytokine production (Fig 2). Notably, while both stimuli at high concentrations induced IL-12 and IL-6, the soluble lysates induced low TNFα and failed to induce IL-10 production at the tested concentrations (Fig 2). Since the activation status of the DCs evidenced by the expression of cell surface markers was comparable at high doses of all the antigenic preparations tested (data not shown), these findings indicate that the presence of different PAMPs plays distinct roles in the process of induction of cytokine release [28–29].

**Fig 3:**
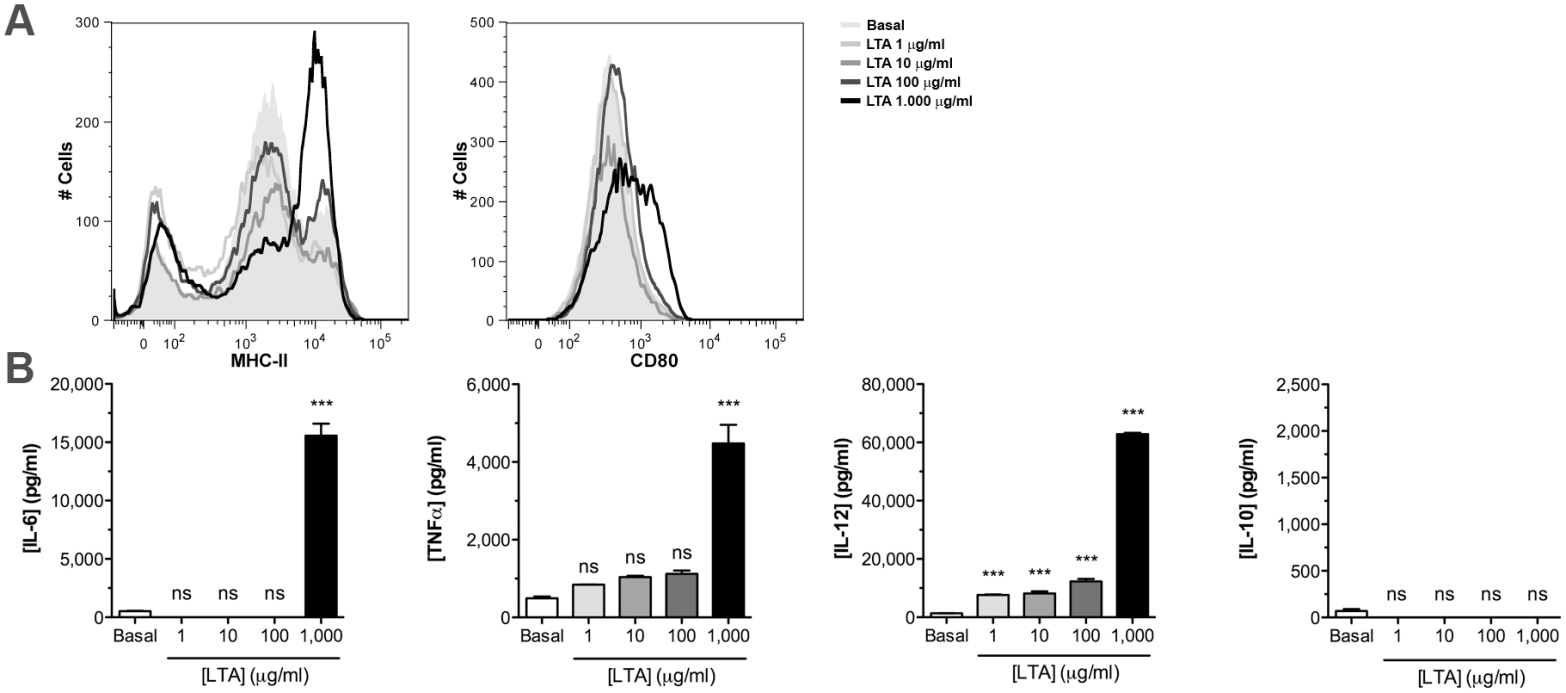
LTA purified from probiotic strain *E*. *faecalis* CECT7121 induce *in vitro* activation of BM-DCs. Lipoteichoic acids (LTA) were purified from *E*. *faecalis* CECT7121, and BM-DCs were stimulated *in vitro* with different concentrations (24 h). Cell surface marker expressions were measured by flow cytometry (A), and cytokines were evaluated in culture supernatants by ELISA (B). Flow cytometry results are representative from 2 independent experiments. Cytokine levels are expressed as mean pg/mL ± SEM. ns: non-significant, ***p<0.001 (One-way ANOVA and Dunnett’s Multiple Comparison tests). LTA from *E*. *faecalis* CECT7121 induce a dose-dependent up-regulation of activation molecules and the secretion of different cytokines by BM-DCs with the exception of IL-10.

Considering that lipoteichoic acids (LTA) are postulated as a main PAMP in the cell wall fraction of Gram positive bacteria, LTA from *E*. *faecalis* CECT7121 was purified and employed to stimulate *in vitro* BM-DCs. LTA induced a dose-dependent up-regulation of classical DC maturation markers such as MHC-II, CD80, CD40, and CD83 (Fig 3A). Moreover, upon stimulation with different concentrations of LTA, BM-DCs secreted different cytokines including IL-6, TNFα, and IL-12 but no IL-10 (Fig 3B). Our results demonstrate that LTA may have major relevance in the stimulatory activity of this probiotic strain.

### BM-DC activation by *E*. *faecalis* CECT7121 is dependent on the presence of MyD88

**Fig 4:**
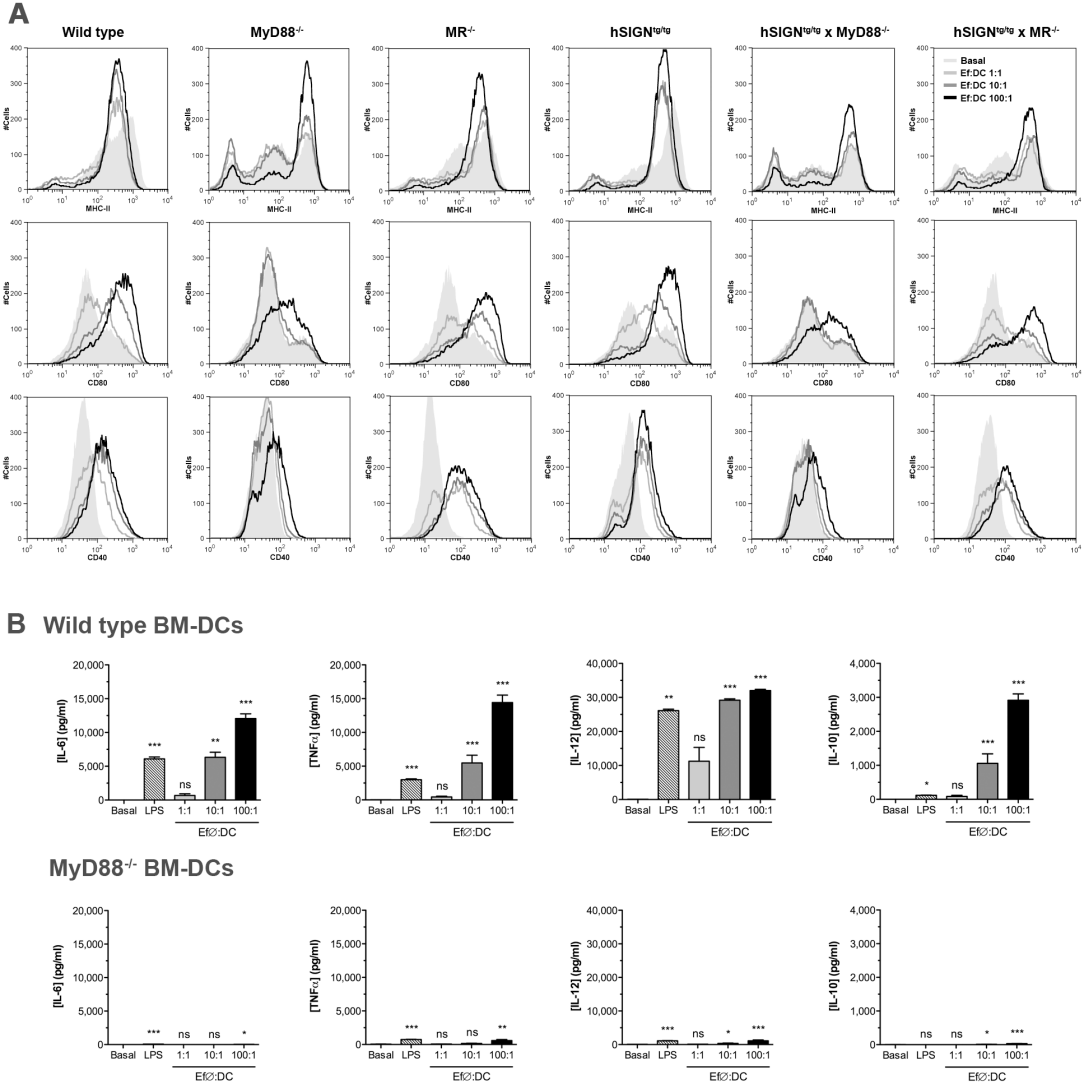
*In vitro* stimulation of genetically modified BM-DCs by *E*. *faecalis* CECT7121. BM-DCs generated from MyD88^-/-^, hSIGN, MR^-/-^, hSIGN × MyD88^-/-^, and hSIGN × MR^-/-^ mice were *in vitro* stimulated with heat-killed *E*. *faecalis* CECT7121 (Ef∅) for 24 h. MHC-II, CD80, and CD40 expression levels were analyzed by flow cytometry (A), and IL-6, TNFα, IL-12, and IL-10 were determined by ELISA (B). Flow cytometry results are representative from 2 independent experiments. Cytokine levels are expressed as mean pg/mL ± SEM. ns: non-significant, *p<0.05, **p<0.01, ***p<0.001 (Kruskal-Wallis ANOVA and Dunn’s Multiple Comparison tests). Absence of MyD88 abrogates BM-DC maturation induced by *E*. *faecalis* CECT7121. Modifications in the expression of lectins such as human DC-SIGN or mannose receptor do not seem to influence DC activation by *E*. *faecalis* CECT7121.

In order to study the role of specific PRRs involved in DC activation by *E*. *faecalis* CECT7121, genetically modified BM precursors were employed. As observed by flow cytometry analysis and cytokine expression, only TLR signaling seemed to be crucial for DC activation by *E*. *faecalis* CECT7121, since absence of MyD88 abrogated BM-DC maturation induced by this probiotic bacterium (Fig 4). Only a high MOI (100:1) induced an up-regulation of MHC-II, CD80, and CD40 expression in MyD88^-/-^ DCs, as well as a low production of IL-12 and TNFα, demonstrating that the major effect of *E*. *faecalis* CECT7121 is mediated via MyD88-dependent pathways [28].

On the other hand, modifications in the expression of different lectins such as human DC-SIGN in transgenic murine hSIGN DCs or mannose receptor knock-out cells do not seem to influence DC activation by *E*. *faecalis* CECT7121 (Fig 4A), indicating that these receptors are not relevant for DC activation as observed by other authors [16, 30–34].

### *In vitro* probiotic-activated BM-DCs drive the polarization of T cells towards a Th1 profile

**Fig 5:**
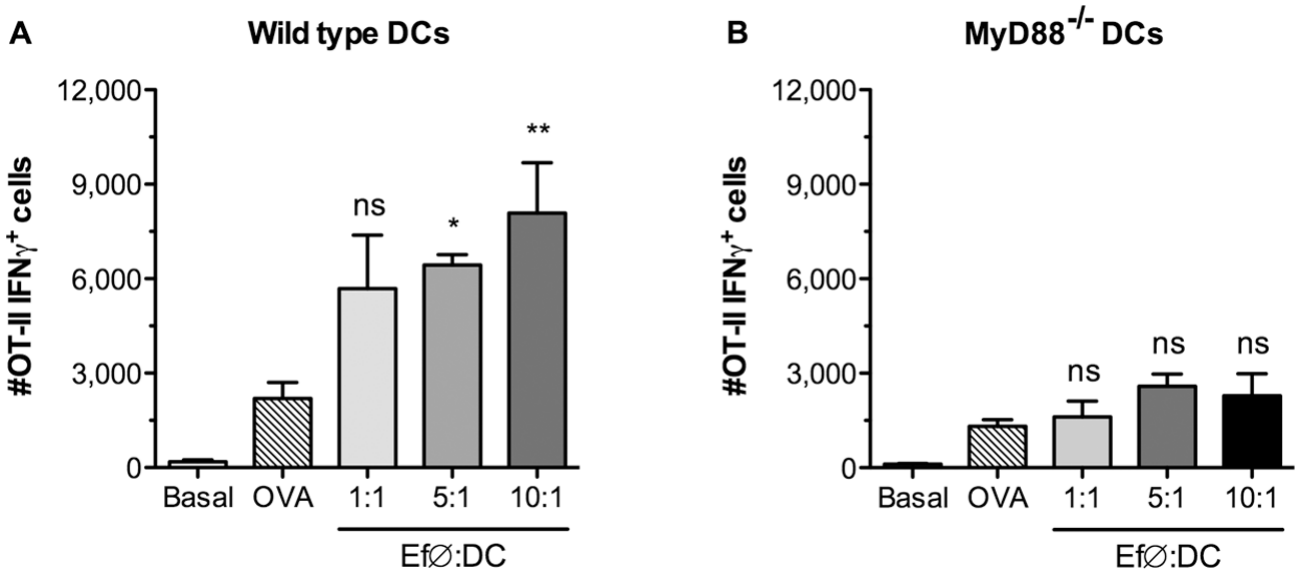
DC-T cell co-culture under stimulation with *E*. *faecalis* CECT7121. BM-DCs pulsed with ovalbumin and further stimulated with heat-killed *E*. *faecalis* CECT7121, were co-cultured with CD4^+^ enriched OT-II T lymphocytes (96 h). After PMA + Ionomycin re-stimulation, CD4^+^IFNγ^+^ cells were analyzed by flow cytometry. Comparisons were performed between OVA and probiotic-stimulated cultures. ns: non-significant, *p<0.05, **p<0.01 (One-way ANOVA and Dunnett’s Multiple Comparison tests). *E*. *faecalis* CECT7121-primed BM-DCs induce the production of IFNγ by CD4+ T cells. This effect is TLR-dependent, since the absence of MyD88 abrogates this induction.

As *E*. *faecalis* CECT7121 was able to fully activate *in vitro* BM-DCs and to induce the release of high levels of IL-12, DC-T cell *in vitro* co-cultures were employed in order to determine the polarizing effects of probiotic-primed DCs. Wild-type and MyD88^-/-^ BM-DCs were stimulated with *E*. *faecalis* CECT7121, pulsed with ovalbumin (OVA), and co-cultured with CD4^+^ T cells purified from naïve OT-II mice. As expected, *E*. *faecalis* CECT7121-primed BM-DCs induced the production of IFNγ by CD4^+^ T cells (Fig 5A) and this effect was TLR-dependent, since the absence of MyD88 abrogated the induction of Th1 phenotype (Fig 5B). These results reinforce the hypothesis that *E*. *faecalis* CECT7121 stimulation induces an immune response skewed towards the production of IFNγ (the hallmark of Th1 responses).

### *E*. *faecalis* CECT7121-primed BM-DCs also induce the production of IFNγ *in vivo* after adoptive cell transference

**Fig 6:**
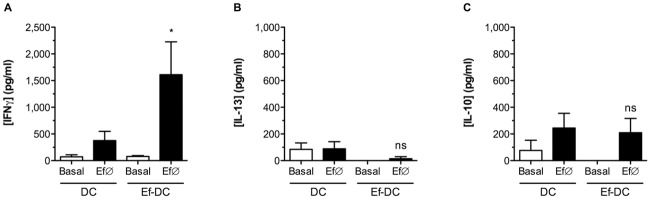
*In vivo* influence of *E*. *faecalis* CECT7121-pulsed DCs, and IFNγ production by spleen cells. C57BL/6 BM-DCs were pulsed with heat-killed *E*. *faecalis* CECT7121 (Ef∅) for 18 h, and then adoptively transferred to naïve C57BL/6 mice. After 7 days, spleen cells were *ex vivo* stimulated with Ef∅ (72 h) and cytokines were analyzed in culture supernatants by ELISA (A, IFNγ; B, IL-13; **C**, IL-10). Cytokine levels are expressed as mean pg/mL ± SEM. Comparisons were performed between DC and Ef-DC groups. ns: non-significant, * *p*<0.05 (Unpaired Student’s *t* test). *E*. *faecalis* CECT7121-primed BM-DCs adoptively transferred to naïve mice induce the specific-secretion of IFNγ by spleen cells without production of IL-13 or IL-10.

DC-T cell co-cultures demonstrated that *E*. *faecalis* CECT7121 activates BM-DCs and promotes the production of IFNγ. To determine whether this effect was relevant *in vivo* and whether DCs were important in this phenomenon, *E*. *faecalis* CECT7121-primed BM-DCs were injected i.p. into naïve C57BL/6 mice. After 7 days, spleen cells from injected animals were stimulated *ex vivo* with Ef∅ and different cytokines were measured in culture supernatants by ELISA. Our results demonstrate that *E*. *faecalis* CECT7121-primed BM-DCs induce the accumulation of specific IFNγ -producing cells *in vivo*, without production of neither IL-13 nor IL-10 (Fig 6A–6C). This observation indicates that DCs are relevant for *E*. *faecalis* CECT7121 immunostimulation *in vivo*, activating the adaptive immune response and inducing the production of IFNγ *in vivo*.

### Intragastric immunization with live *Enterococcus faecalis* CECT7121 does not modify the total cellularity at local or systemic levels

In order to analyze the *in vivo* effects of *E*. *faecalis* CECT7121, BALB/c mice were i.g. inoculated with this probiotic strain during 3 consecutive days as previously described [12]; this immunization schedule ensures *E*. *faecalis* CECT7121 implantation at least for 18 days, without modifying the commensal microflora. In the present work, we studied several immunological parameters on days 4 and 11 after the last inoculation, a period during which the bacterium still remains implanted in the murine intestine.

After immunization, no significant changes in the total cellularity were observed neither in the spleen nor in the MLNs of *E*. *faecalis* CECT7121-treated mice compared with non-treated control group (Table 1). Then, we characterized different cell populations by flow citometry using antibodies that specifically recognized CD3ε, CD4, CD8α, CD19, CD45R/B220, CD11c, CD11b, and Gr-1, markers of T cells, B cells, and myeloid cells. No significant changes were detected in any of these cell populations tested on day 4 or 11 post-administration, with the exception of a marginal, non-significant increment of total T lymphocytes (CD3ε^+^ cells) (Table 2). This increase in T cells was mostly due to a rise in CD4^+^ but not CD8α^+^ cells in the spleen and especially in the local MLNs (Table 3).

**Table 1:** Total number of spleen and MLN cells after i.g. treatment with *E*. *faecalis* CECT7121.

Total cell number (x10^6^; mean ± SEM)			
	PBS	Day 4	Day 11
Spleen	161.50 ± 14.73	147.50 ± 12.36	152.90 ± 17,83
MLNs	27.37 ± 2.83	30.24 ± 4.06	23.63 ± 1.56

Total cell numbers in the spleen and MLNs of PBS and *E*. *faecalis* CECT7121-treated BALB/c mice were determined by counting in Neubauer’s haemocytometer. Results are from 2 independent experiments.

**Table 2:** Spleen immune cell populations after *in vivo* treatment with *E*. *faecalis* CECT7121.

Spleen cell populations (%; mean ± SEM)			
	PBS	Day 4	Day 11
CD3ε^+^	33.82 ± 2.85	42.97 ± 3.36^ns^	38.90 ± 2.90^ns^
CD19^+^ B220^+^	40.32 ± 2.75	41.17 ± 4.95	42.68 ± 3.63
CD11b^+^	6.71 ± 1.14	4.45 ± 0.58	4.99 ± 0.80
CD11c^+^	1.29 ± 0.32	0.67 ± 0.04	1.59 ± 0.05
Gr-1^+^	2.22 ± 0.20	2.00 ± 0.16	1.74 ± 0.22

Cellular populations in the spleen of control and immunized mice were determined by flow cytometry. Results are from 2 independent experiments. ^ns^non-significant (One-way ANOVA plus Dunnett’s Multiple Comparison tests).

**Table 3:** CD4^+^ and CD8α^+^ cells in spleen and MLNs after probiotic immunization.

CD4 and CD8α cells (%; mean ± SEM)				
		PBS	Day 4	Day 11
Spleen	CD4^+^	19.57 ± 1.74	21.74 ± 1.44^ns^	23.18 ± 1.67^ns^
	CD8α^+^	7.84 ± 1.31	7.95 ± 0.91	7.57 ± 1.33
	Double negative	72.48 ± 3.00	70.24 ± 1.83	69.16 ± 2.17
MLNs	CD4^+^	48.27 ± 5.10	55.26 ± 2.35^ns^	55.08 ± 1.52^ns^
	CD8α^+^	16.45 ± 2.08	17.52 ± 1.46	16.00 ± 1.73
	Double negative	35.05 ± 6.51	27.02 ± 2.98	28.74 ± 1.70

Sub-populations of T lymphocytes in the spleen and MLNs of control and probiotic-treated animals were determined by flow cytometry. Results are from 2 independent experiments. ^ns^non-significant (One-way ANOVA plus Dunnett’s Multiple Comparison tests).

### Treatment with the probiotic strain primes T cell proliferation and production of IFNγ

**Fig 7:**
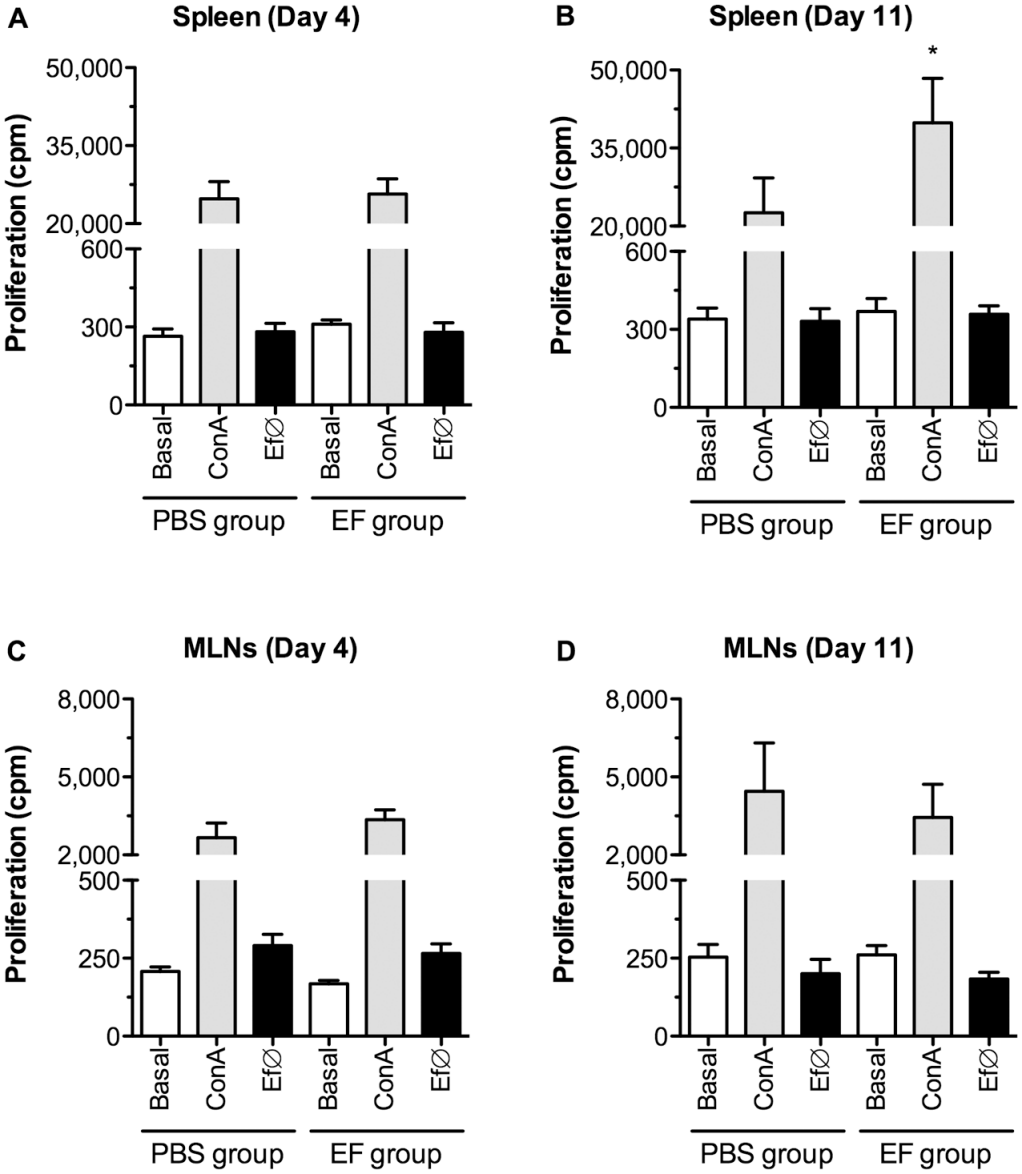
Spleen and MLN cells proliferation of probiotic-treated animals. Spleen (A and B) and MLNs (C and D) cells were *ex vivo* stimulated with ConA and heat-killed *E*. *faecalis* CECT7121 (Ef∅), and proliferation was assessed by ^3^H-thymidine uptake. Results were expressed as mean cpm ± SEM from 2 independent experiments, and comparisons were performed between PBS and EF groups. *p<0.05 (Mann-Whitney U-test). In both spleen and MLNs cell cultures, cells obtained from treated mice compared to control animals show an increase in ConA-induced proliferation on day 11 post-treatment without specific proliferation.

For a further evaluation the influence of *E*. *faecalis* CECT7121 on the immune system at the local and systemic levels, T cell responses were analyzed *ex vivo* after polyclonal (ConA) or specific (Ef∅) stimulation. In both spleen and MLNs cell cultures, cells obtained from treated mice showed similar basal proliferation compared to cells obtained from control animals (Fig 7). Nevertheless, *in vivo* immunization with *E*. *faecalis* CECT7121 exerted a 1.8 fold increase in ConA-induced *ex vivo* spleen cell proliferation on day 11 post-treatment (Fig 7A and 7B), demonstrating a systemic stimulatory effect of this probiotic on the immune system. Additionally, Ef∅ was employed as stimulus to evaluate specific spleen cell proliferation, but no differences were observed between experimental and control groups. Furthermore, no differences were detected in MLN cells proliferation (Fig 7C and 7D). Correlating with the T-cell proliferation observed for both groups of animals, only cells stimulated with ConA secreted high levels of IL-2, a key cytokine for lymphocyte proliferation (data not shown).

**Fig 8:**
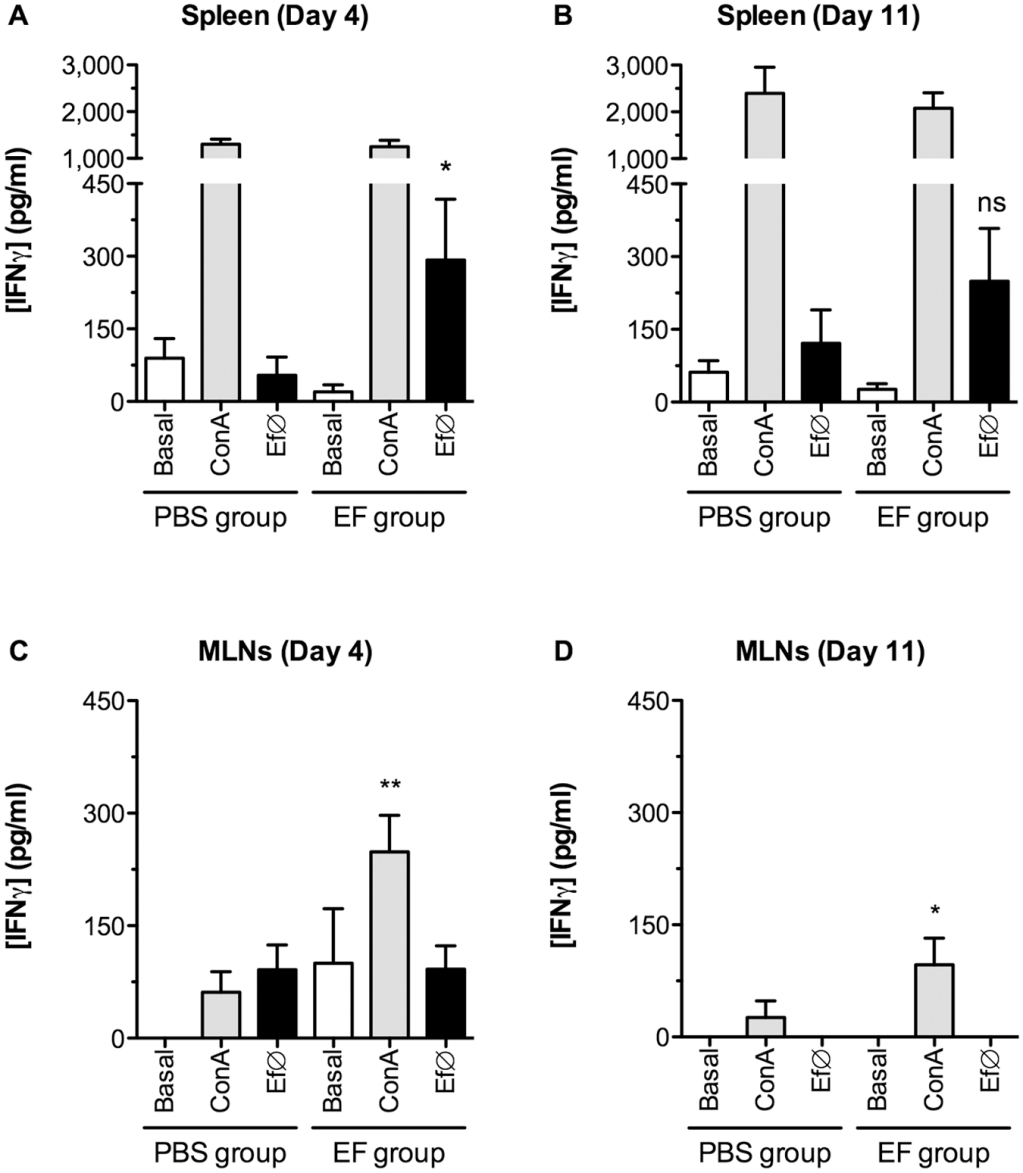
IFNγ production after *in vivo* immunization with *E*. *faecalis* CECT7121. Supernatants from spleen (A and B) and MLNs (C and D) cell cultures were analyzed by direct ELISA to determine the levels of IFNγ. Results were expressed as mean pg/mL ± SEM from 2 independent experiments, and comparisons were performed between PBS and EF groups. ns: non-significant, *p<0.05, **p<0.01 (Mann-Whitney U-test). The inoculation of *E*. *faecalis* CECT7121 induces a rapid IFNγ production at local (MLNs) and systemic (spleen) levels.

We next analyzed the production of different cytokines after i.g. inoculation with *E*. *faecalis* CECT7121 and observed a rapid IFNγ production by spleen cells stimulated with Ef∅ as observed on day 4 post-treatment (5.4 fold increase; Fig 8A). The production of IFNγ was no longer statistically relevant on day 11, although the same tendency was still observed (Fig 8B). Overall, these results are in concordance with those obtained in our previous studies where *E*. *faecalis* CECT7121 induced a Th1-skewed systemic response [12–15].

In MLNs, cells from immunized mice also produced higher amounts of IFNγ after polyclonal stimulation with ConA on days 4 and 11 post-treatment (4.0 fold increase in both cases), although on day 11 the levels were lower (Fig 8C and 8D). Contrarily, no IFNγ secretion was detected upon specific stimulation with Ef∅. Altogether, these results show that the i.g. treatment with this probiotic exerts an immunostimulatory effect, by transiently increasing the production of IFNγ both at local and systemic levels.

Apart from IFNγ, spleen cells from immunized mice showed a slight but significant increase in the production of IL-6 after ConA stimulation (1.8 fold increase on day 4; Control 39.04 pg/mL ± 14.69 *vs* EF 70.07 pg/mL ± 13.84, *p<0.05). Other cytokines tested (IL-12, IL-2, and IL-13) did not show any significant change associated with the immunization with *E*. *faecalis* CECT7121 (data not shown), at least on the tested time-points.

### The probiotic strain *E*. *faecalis* CECT7121 does not modify the recruitment of regulatory cell populations

**Fig 9:**
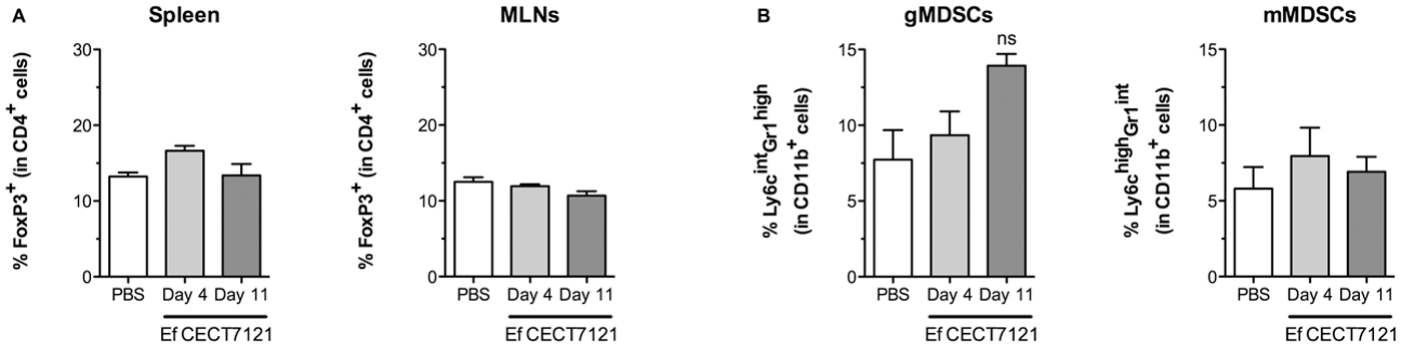
Evaluation of regulatory cells in the spleen of probiotic-immunized animals. Spleen and MLNs cells were stained employing specific antibodies to determine CD4^+^FoxP3^+^ Tregs by flow cytometry (A). CD11b^+^Ly6C^int^Gr-1^high^ gMDSCs and CD11b^+^Ly6C^high^Gr-1^int^ mMDSCs were also determined by flow cytometry in the spleen of PBS and immunized mice (B). Results were expressed as mean cpm ± SEM from 2 independent experiments, and comparisons were performed between PBS and probiotic-treated mice. ns: non-significant (One-way ANOVA and Dunnett’s Multiple Comparison tests). The inoculation of *E*. *faecalis* CECT7121 does not induce Tregs accumulation neither in spleens nor in MLNs, but causes a non-significant accumulation of gMDSCs in the spleen of immunized mice.

Different myeloid and lymphoid cell populations actively regulate the immune response in order to avoid exacerbated reactions that may harm our body. It has been shown that certain probiotic strains can exert their functions by activating regulatory cells [35]. We therefore analyze changes in two main regulatory cell populations, including Tregs (CD4^+^FoxP3^+^) and monocytic and granulocytic myeloid-derived suppressor cells (mMDSCs: CD11b^+^Ly6C^high^Gr-1^int^; gMDSCs: CD11b^+^Ly6C^int^Gr-1^high^) after *E*. *faecalis* CECT7121 treatment. While no Tregs accumulation was observed neither in spleens nor in MLNs, a non-significant tendency in the accumulation of gMDSCs was observed in the spleen of immunized mice (day 11 post-immunization) (Fig 9A and 9B).

Altogether, these results allow us to conclude that the effects of *E*. *faecalis* CECT7121 are not mediated by regulatory cell populations.

## Discussion

The gastrointestinal microbiota is essential to human health, as it contributes to food digestion and to the development and optimal functioning of the intestinal immune system [1]. These properties could be exploited in the modulation of both local and systemic immune responses.

*Enterococcus faecalis* CECT7121 is a probiotic strain that has been demonstrated to implant itself, persist, and induce protective immune responses in several biological models [12–15]. This work was aimed at elucidating the immunological mechanisms exerted by *E*. *faecalis* CECT7121 in immunocompetent mice.

DCs are important in the instruction of both innate and adaptive responses and their activation by probiotics generally modulate the immune response towards a Th1-phenotype or a T regulatory profile [36–38]. Herein, we evaluated DCs as a possible target for probiotic stimulation. Heat-killed *E*. *faecalis* CECT7121 probiotic strain induced a strong dose-dependent secretion of IL-12, IL-6, TNFα, and IL-10 on BM-DCs, as well as an increased expression of activation markers such as MHC-II, CD80, and CD40. It is well known that IL-12 and IFNγ induce Th1-responses, while IL-4 predominantly generates Th2-profiles, and IL-6 and TGFβ drive the generation of Th17 cells [39–40]. Moreover, the production of IL-10 by some probiotic strains may induce the generation of Treg cells [39–41]. In this context, the stimulation of DCs by *E*. *faecalis* CECT7121 results in a variety of cytokines that drive phenotypical and functional changes during T-cell activation, which will in turn define the fate of T-cell responses. Our results suggest that *E*. *faecalis* CECT7121 may drive the activation of T cell responses and the production of Th1-responses, probably due to the high levels of IL-12 secreted by stimulated DCs. On the other hand, we also detected high IL-10 secretion when BM-DCs were stimulated with our probiotic strain. Although it is known that stimulation of DC via TLRs may trigger IL-10 production when accompanied by activation of other PRRs such as lectins [29], the high levels of this anti-inflammatory cytokine produced *in vitro* suggest that this mechanism may have an important role *in vivo*. In summary, DCs seem to be an important cellular target of *E*. *faecalis* CECT7121 as it does not only trigger inflammatory IL-12/IFNγ-responses, but also induces the simultaneous production of anti-inflammatory molecules.

MyD88-KO DCs showed the relevance of TLR signaling during *E*. *faecalis* CECT7121 stimulation: the abrogation of this adapter protein impaired DC maturation and cytokine production. In addition, the general importance of lectin-signaling pathways in the shaping of DCs functions has been extensively analyzed in the past [4,8,29]; in this sense, we also studied the influence of two different lectin receptors: DC-SIGN and MR. Neither the expression of human DC-SIGN nor the absence of MR on BM-derived murine DCs seemed to modify the stimulatory effect of this probiotic. Nevertheless, the importance of lectin-signaling pathways should not be ruled out, since there is a bulk of evidence indicating the influence of these signals in the shaping of DC functions [29]. In addition to the study of receptors involved in the recognition of *E*. *faecalis* CECT7121, different antigenic preparations isolated from this probiotic induced different cytokine responses, with LTA being an interesting immunostimulatory molecule since it successfully activated DCs *in vitro* and induced the production of all pro-inflammatory cytokines tested. Gram-positive LTAs have gained interest as important immune modulators based on several studies, both *in vitro* and *in vivo* [1,42].

To confirm the importance of DC stimulation by this probiotic on T cell responses, different experiments both *in vitro* (pulsed DC-T cells co-cultures) [3,43] and *in vivo* (IFNγ-producing responses by probiotic stimulated-DCs adoptive transfer) [24–27] proved that the activation of DCs by *E*. *faecalis* CECT7121 leads to the generation of Th1-adaptive cellular responses, most likely because of the high levels of IL-12 produced after the probiotic stimulation.

During *in vivo* studies after the inoculation of *E*. *faecalis* CECT7121, although no changes in the total number of spleen or MLN cells were observed, this probiotic caused an enhancement of local and systemic immunity, inducing higher proliferative responses and skewing the immune response towards the production of IFNγ. This IFNγ production was higher on day 4 and diminished by day 11 after treatment, showing signs of a kinetic response taking place both at local and systemic levels. This dynamic response indicates the usefulness of this probiotic bacterium to direct specific responses towards Th1-profiles, making it an interesting candidate to be used as oral adjuvant [13]. Other authors have demonstrated that the ingestion of certain probiotic strains may induce high proliferative responses of both T and B cells [44–45]. While this immune activation influences polyclonal, *E*. *faecalis* CECT7121 non-specific responses, the lack of specific proliferation in both lymphoid organs could be in line with the capacity of probiotic bacteria to behave as a commensal microorganism without inducing uncontrolled inflammatory responses. Regulatory mechanisms in the gut are triggered in order to avoid inflammatory responses against commensal microflora. In fact, it has been demonstrated that gut-associated lymphoid tissues DCs induce local protective IgA adaptive responses against commensal bacteria as an important regulatory mechanism [46]. Indeed we have initially observed an increase in total IgA+ cells in the lamina propria after the administration of *E*. *faecalis* CECT7121 at time-points as early as 1 day after the last inoculation (unpublished data). Probably, this rapid expansion of IgA+ clones could be the consequence of a T-cell independent IgA class switch as it has been demonstrated that DCs that have sampled endogenous flora induce IgA production through T-dependent and independent mechanisms [47]. Further studies are required to understand the mechanisms triggered by this probiotic strain at the intestinal level. Indeed, it would be interesting to study whether *E*. *faecalis* CECT7121 is able to increase luminal ATP levels as some commensal bacteria and thus promote T cell differentiation as shown previously Atarashi *et al* [48]. Another explanation for the lack of specific proliferation responses could be related to a direct inhibition of T and/or B cell proliferation. Plitnick *et al* have demonstrated that LTA from Gram-positive bacteria can specifically bind to IL-2 and impair the mononuclear cells proliferation induced by tetanus toxoid stimulation [49]. Moreover, these authors have also described a dose-dependent production of IFNγ after LTA stimulation, not only demonstrating that the inhibitory effect on IL-2 seems to be selective, but also that this PAMP *per se* is able to induce the secretion of IFNγ and could also be responsible for our observations regarding the production of this cytokine.

Taking into account immune regulatory mechanisms, a non-significant tendency in the splenic accumulation of granulocytic MDSCs could constitute a mechanism to prevent exacerbated inflammatory responses, at least during the time span studied. Although MDSCs have been primarily associated with cancer, many studies have demonstrated their role in bacterial, viral, and even parasitic infections [50–53]. Regarding Tregs accumulation, some probiotics usually associated to *Lactobacillus* strains are able to increase the number of CD4^+^CD25^+^FoxP3^+^ cells both at local and systemic lymphoid organs [54]. To our knowledge, there are no reports that link Tregs accumulation and *E*. *faecalis* strains, and our results prompt us to conclude that this probiotic does not affect the generation and recruitment of Tregs, at least at the time-points tested after intragastric immunization.

Considering the rapid *in vivo* production of IFNγ after *E*. *faecalis* CECT7121 inoculation, Hua and colleagues obtained similar results when they studied the secretion of different cytokines by mononuclear cells and DC cultures from healthy donors that consumed a commercial mix of probiotics [55]. These authors found that the IFNγ levels were higher in the treated group, and that this increase was notably dependent on the ratio of bacteria used as stimulus. These results are in line with our observations, especially taking into account that the commercial mix employed by Hua *et at* includes an *Enterococcus faecalis* probiotic strain. Additionally, Kosaka and colleagues have described that the ingestion of *Lactococcus lactis* subsp. *cremoris* FC induces the production of IFNγ and IL-10 by spleen cells; these effects also seem to be due to the activation of DCs in a MyD88-dependent manner, and the subsequent production of IL-12 and IL-18 [56].

To sum up, our results demonstrate that the immune stimulation triggered by *E*. *faecalis* CECT7121 involves the activation of the innate response (being DCs a key target) and the consequent development of Th1-related, cellular adaptive responses. Probably, the humoral immune response will be also modulated by the ingestion of *E*. *faecalis* CECT7121, but this aspect was not studied in this work. This work globally analyzes for the first time different aspects of an *Enterococcus faecalis* probiotic strain and allows concluding that this bacterium is suitable for enhancing immune responses and skewing the cytokine profile to the production of IFNγ. *Enterococcus faecalis* CECT7121 may be employed as a novel adjuvant strategy, both for oral or systemic vaccination.
